# Random Pattern Vertically Oriented, Partial Thickness Buccinator Myomucosal Flap for Intraoral Reconstruction:A Report of Two Cases

**Published:** 2016-05

**Authors:** Amin Rahpeyma, Saeedeh khajehahmadi

**Affiliations:** 1 *Oral and Maxillofacial Diseases Research Center, Oral and Maxillofacial Surgery ,School of Dentistry, Mashhad University of Medical Sciences, Mashhad, Iran. *; 2 *Dental Research Center ,Oral and Maxillofacial Pathology , School of Dentistry, Mashhad University of Medical Sciences, Mashhad, Iran. *

**Keywords:** Artery, Flap, Mandibular Reconstruction

## Abstract

**Introduction:**

Reconstruction of the oral cavity with a flap design containing the buccal mucosa and buccinator muscle but excluding the facial artery and vein is the topic of these case reports.

**Case Reports:**

This article uses random pattern vertically oriented partial thickness buccinator myomucosal flap for intraoral reconstruction in two cases. The first was for lining the mandibular anterior vestibule in a trauma patient. The second was for oral side coverage of bone graft in special cleft patient. In both patients, this flap survived and good bone coverage with non-keratinized mucosa was obtained.

**Conclusion:**

Thin long buccal myomucosal flap not including facial artery and vein can survive.

## Introduction

Reconstruction of a resected oral mucosa with skin flaps has some drawbacks including bad odor, color mismatch, over contouring, and dryness. Pedicled buccal mucosal flap has the advantage of replacing the lost tissue with the same type of tissue; however, it is limited in its length. Including the buccinator muscle in these flaps changes it structurally from a mucosal to a myomucosal flap; however, there is the advantage of more blood supply to the flap. FAMM (facial artery musculomucosal) flap was introduced by Pribaz in 1992 (1). It is an obliquely oriented intraoral cheek flap, anterior to the stensen’s duct, and contains buccal mucosa, buccinator muscle, and facial artery and vein(2). It is a pedicled flap that can be superiorly or inferiorly based (3). Inferiorly based FAMM flap is an axial pattern flap which is supplied by the facial artery and vein. Superiorly based FAMM flap has reverse flow and is nourished by the angular artery (4,5). In literature, including the facial artery in the flap is insisted upon in order to guarantee blood perfusion (6). Including the facial vein is advised but not mandatory for flap survival. Venous drainage through vasovasorum surrounding the facial artery and submucosal plexus in the flap pedicle, is sufficient (7,8).This article attempts to show that myomucosal flap with the same paddle design as the FAMM flap but not including the facial artery and vein can survive.

***Surgical Technique***

In the buccal mucosa anterior to the stensen’s duct and one centimeter behind the oral commissure, a vertically oriented myomucosal flap not including the facial artery and vein was prepared. Partial thickness of the buccinator muscle was raised within the flap. This flap may be superiorly or inferiorly based, depending on the location of the defect.

## Case Reports

**Fig 1:**
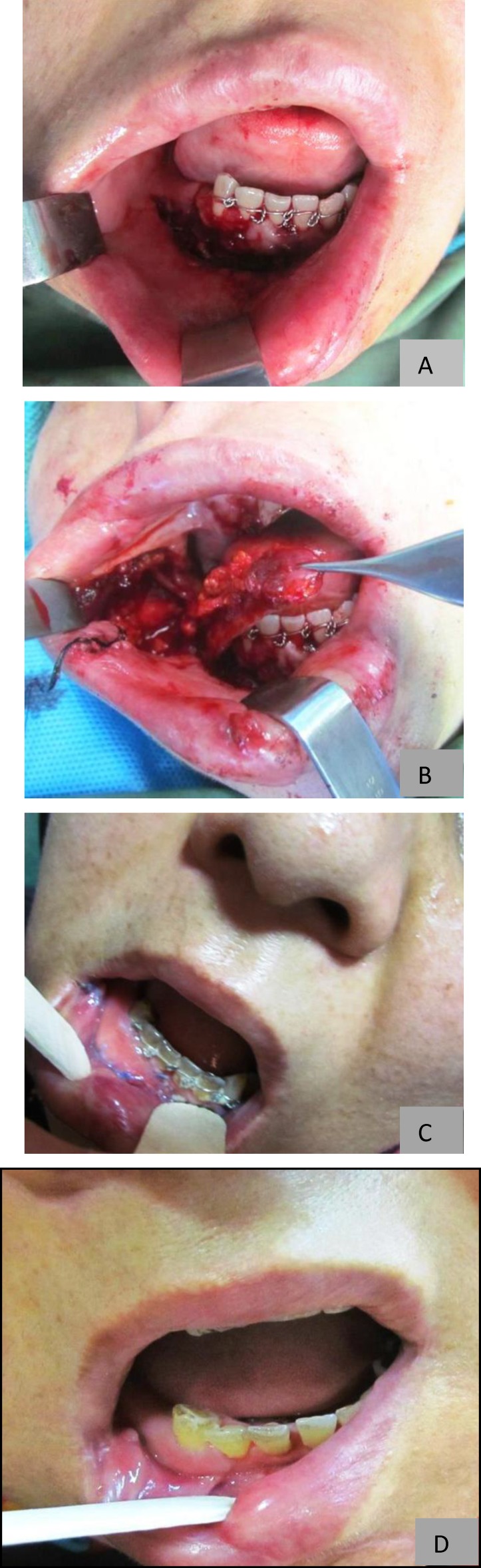
a) Anterior mandibular fracture and avulsed labial vestibular mucosa b) Inferiorly based flap after harvesting. c) Photograph taken three weeks after operation before pedicle division. d) Post-operative result three month after operation

*Case 1:* The first patient was a 42-year-old female with an anterior mandibular dentoalveolar fracture. Avulsion of the labial mucosa covering the vestibule and lingualy displaced fractured dentoalveolar segment was present. After proper reduction of the segment and splinting with a wire to the adjacent teeth, the lost labial mucosa was replaced with an inferiorly based partial thickness pedicled buccinator myomucosal flap, which was not containing a vertically oriented named artery or vein (Fig. 1). Three weeks later the pedicle was divided.

*Case 2:* The second patient was a 28-year-old female patient with bilateral cleft lip/alveole , which had been operated during infancy, with the wrong concept of premaxillectomy. There was a large oronasal fistula present behind the upper lip.

**Fig 2:**
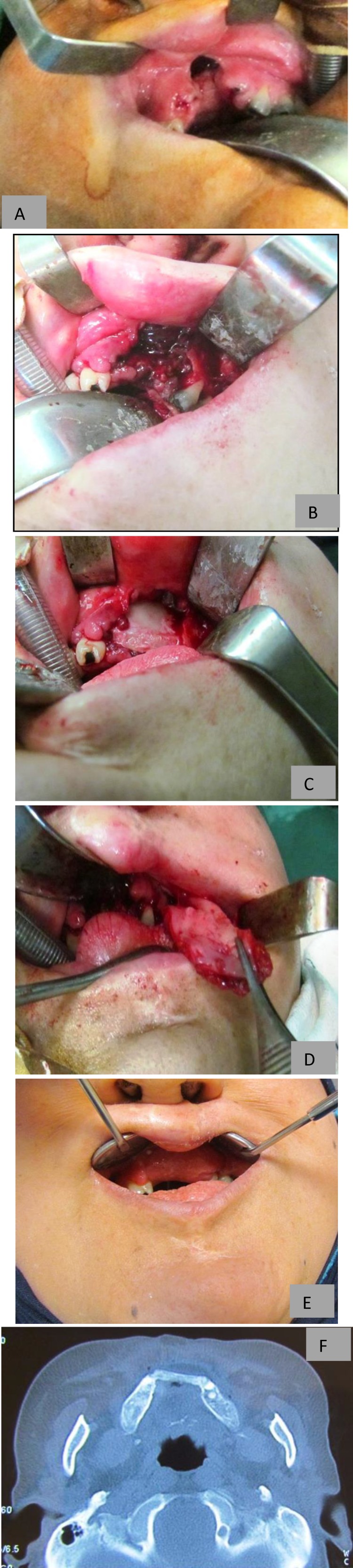
a) Permaxillectomy defect and large oronasal fistula. b) Inferior turbinate flap is used for nasal side closure. c) Bone graft wedged in between the lateral segments. d) Superiorly based flap is used for bone graft coverage. e) Photograph taken three month after operation. f) CBCT shows position of the bone graft in between lateral segments

The nasal floor was reconstructed with the aid of an anteriorly based inferior turbinate flap and a bone graft was obtained from the anterior iliac crest using a medial approach. Monocortical (coticocancellous) bone block was wedged between the two lateral segments and firmly remained stable without the need for internal fixation devices such as a miniplate. Superiorly based partial thickness pedicled buccinator myomucosal flap not including the facial artery and vein was used for bone graft coverage. The flap pedicle was divided three weeks after the operation (Fig.2).

## Discussion

The buccinator muscle is nourished by the maxillary and facial arteries and is covered by the buccal mucosa. Different type of flaps are designed in the buccal region ranging from a buccal transposition flap that has random circulation to axial pattern flaps with leadership of FAMM flap. Vertically oriented, partial thickness buccinator myomucosal flap, which is the topic of this article, falls in between these two categories of flaps. Random flaps based on buccal mucosa without supporting buccinator muscle need a wider base and have limited length (9).

FAMM flap is an axial pattern flap and an arterialized flap (10). Including the facial artery in flap thickness guarantees its blood perfusion. Paddle configuration dictates the course of facial vessels. Following the predetermined course of the facial artery by color Doppler is possible but not necessary for flap survival.In true FAMM flaps, the facial artery should be ligated at the flap tip and should be included in the flap base. Increasing the flap thickness and including the full thickness buccinator muscle, in addition to the need for deep dissection, are limitations of this flap for capturing the facial artery in flap thickness.

Oral mucosa has a rich blood supply, so a random pattern flap, which contains partial thickness of the buccinator muscle, can survive. Flap perfusion depends on pedicle width for arterial circulation and venous drainage. From a surgical point of view, it is simpler surgery and flap thickness is less but with shorter pedicle length in comparison with true FAMM flap.

The rule of the length:width ratio, which is normally 3:1 in random pattern flaps, should be strictly adhered (11).

## Conclusion

Buccinator myomucosal flap with the same paddle design as the FAMM flap but not including the facial artery and vein can survive.
